# Interfacial Coupling SnSe_2_/SnSe Heterostructures as Long Cyclic Anodes of Lithium‐Ion Battery

**DOI:** 10.1002/advs.202204671

**Published:** 2022-11-18

**Authors:** Wang Feng, Xia Wen, Yuzhu Wang, Luying Song, Xiaohui Li, Ruofan Du, Junbo Yang, Hui Li, Jun He, Jianping Shi

**Affiliations:** ^1^ The Institute for Advanced Studies Wuhan University Wuhan 430072 P. R. China; ^2^ Key Laboratory of Artificial Micro‐ and Nano‐structures of Ministry of Education School of Physics and Technology Wuhan University Wuhan 430072 P. R. China

**Keywords:** high‐specific capacity, interfacial coupling, lithium‐ion batteries, long cyclic stability, SnSe_2_/SnSe heterostructures

## Abstract

Tin selenide (SnSe_2_) is considered a promising anode of the lithium‐ion battery because of its tunable interlayer space, abundant active sites, and high theoretical capacity. However, the low electronic conductivity and large volume variation during the charging/discharging processes inevitably result in inadequate specific capacity and inferior cyclic stability. Herein, a high‐throughput wet chemical method to synthesize SnSe_2_/SnSe heterostructures is designed and used as anodes of lithium‐ion batteries. The hierarchical nanoflower morphology of such heterostructures buffers the volume expansion, while the built‐in electric field and metallic feature increase the charge transport capability. As expected, the superb specific capacity (≈911.4 mAh g^−1^ at 0.1 A g^−1^), high‐rate performance, and outstanding cyclic stability are obtained in the lithium‐ion batteries composed of SnSe_2_/SnSe anodes. More intriguingly, a reversible specific capacity (≈374.7 mAh g^−1^ at 2.5 A g^−1^) is maintained after 1000 cycles. The internal lithium storage mechanism is clarified by density functional theory (DFT) calculations and in situ characterizations. This work hereby provides a new paradigm for enhancing lithium‐ion battery performances by constructing heterostructures.

## Graphical abstract

A facile and high‐throughput wet chemical strategy for the batch synthesis of SnSe_2_/SnSe heterostructures is developed and used as anodes of lithium‐ion batteries. In view of the unique hierarchical nanoflower morphology, built‐in electric field, and metallic feature in such heterostructures, excellent specific capacity, high rate performance, and outstanding cyclic stability are achieved in the lithium‐ion battery.

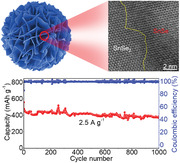

## 1 Introduction

The lithium‐ion battery is considered one of the greatest technological inventions to meet the growing demand for electricity grids due to its high energy density and long cyclic stability.^[^ ^1^, ^2^, ^3^, ^4^, ^5^, ^6^, ^7^, ^8^, ^9^, ^10^, ^11^, ^12^ ^]^ As an important component of the lithium‐ion battery, the anode material has attracted extensive attention.^[^ ^13^, ^14^, ^15^, ^16^, ^17^ ^]^ Therein, tin (Sn) and tin‐based compounds have been widely investigated due to their high theoretical capacities.^[^ ^18^, ^19^, ^20^, ^21^, ^22^, ^23^ ^]^ For example, in view of the layered structure, tunable interlayer space, and abundant active sites, the theoretical capacity of SnSe_2_ is calculated to be ≈813.0 mAh g^−1^.^[^ ^24^ ^]^ Nevertheless, the relatively low electronic conductivity of semiconducting SnSe_2_ inevitably reduces the ion transport efficiency and limits practical applications. In addition, considerable volume expansion (≈400%) is also detected in Sn‐based anodes during the lithiation/delithiation processes, which results in inferior cyclic stability.^[^ ^25^, ^26^, ^27^ ^]^

To solve these problems, great efforts have been devoted, such as incorporating conductive matrices, modulating microstructures, and increasing interlayer distances, to enhance the electronic conductivity and decrease the volume expansion.^[^ ^24^, ^28^, ^29^ ^]^ Nevertheless, the lithium‐ion battery performances of SnSe_2_ are still unsatisfactory. Notably, the energy applications of transition‐metal dichalcogenides (TMDCs) heterostructures have been widely explored,^[^ ^30^, ^31^, ^32^ ^]^ because the large specific surface area reduces the volume expansion and the built‐in electric field improves the charge transport capability.^[^ ^33^, ^34^, ^35^, ^36^, ^37^, ^38^, ^39^, ^40^, ^41^, ^42^ ^]^ For instance, SnS/SnO_2_ heterostructures were synthesized and exhibited excellent charge‐transfer capability.^[^ ^43^ ^]^ The interfacial effect in SnS/SnO_2_ induced an electric field within the nanocrystal, which reduced the ion‐diffusion resistance and facilitated the electron transport efficiency. 2D SnSe_2_/graphene heterostructures were also obtained by the hydrothermal method. The chemical coupling was formed between graphene and SnSe_2_, which buffered the volume variation of the electrode.^[^ ^44^ ^]^ In addition, the metallic property was proposed in SnSe_2_/SnSe heterostructures through the interfacial charge transfer, which enhanced the electronic conductivity significantly.^[^ ^45^ ^]^ Chueh and collaborators synthesized high‐quality SnSe_2_ and SnSe films as well as their vertical heterostructures on glass substrates by the plasma‐assisted chemical vapor reaction method and obtained intrinsic thermal conductivity.^[^ ^46^ ^]^ Nevertheless, the lithium‐/sodium‐ion battery applications of SnSe_2_/SnSe heterostructures are still unaddressed.

Here we develop a facile and high‐throughput wet chemical method to synthesize SnSe_2_/SnSe heterostructures. The superiorities of SnSe_2_/SnSe as the anode of a lithium‐ion battery can be summarized as follows: 1) the hierarchical nanoflower morphology reduces the volume expansion during the charging/discharging processes; 2) the built‐in electric field contributes to the charge transport; 3) the metallic feature improves the electronic conductivity. Accordingly, the remarkable specific capacity, good rate performance, and excellent cyclic stability are obtained in the lithium‐ion battery composed of SnSe_2_/SnSe anode. This work provides an innovative strategy for enhancing lithium‐ion battery performances by building heterostructures, which enables further applications in energy‐related fields.

## 2 Results and Discussion

**Figure 1:**
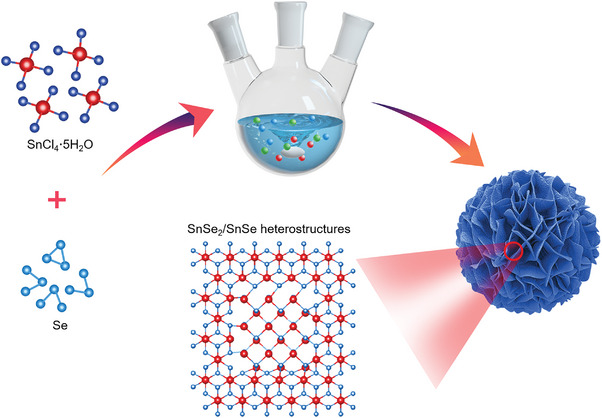
Schematic diagram of the wet chemical synthesis process. The SnCl_4·_5H_2_O and Se powders are used as the precursors and the low boiling point TOA is employed to promote the formation of SnSe_2_/SnSe heterostructures with large specific surface areas.

In view of the batch production feature, the wet chemical method is selected to synthesize SnSe_2_/SnSe heterostructures, as illustrated in **Figure** **1**. SnCl_4_·5H_2_O and Se powders are used as the precursors and then dispersed in n‐octylamine (TOA) and oleylamine (OAm) solutions. During the synthesis process of SnSe_2_/SnSe heterostructures, the TOA plays pivotal roles: 1) as a type of low boiling point ligand and solvent, TOA increases the interaction between the metal precursor and ligand, stabilizes the edge facet, and inhibits the lateral growth of products, which contribute to the self‐assembly and the formation of large specific surface area, and also reduce the volume expansion during the charging/discharging processes;^[^ ^47^ ^]^ 2) TOA modulates the reduction ability of the solution to metal ions during the high‐temperature reaction process, which promotes the formation of SnSe_2_/SnSe heterostructures.^[^ ^48^ ^]^

**Figure 2:**
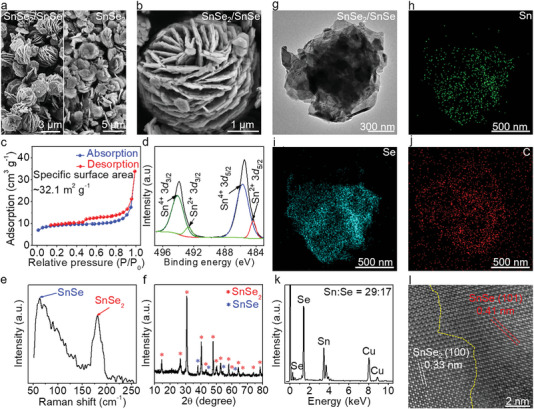
Multiscale determination of SnSe_2_/SnSe heterostructures. a) SEM images of SnSe_2_/SnSe heterostructures and single‐phase SnSe_2_ nanosheets. b) Magnified SEM image of SnSe_2_/SnSe, revealing a unique hierarchical nanoflower morphology. c) Nitrogen adsorption/desorption isotherms of SnSe_2_/SnSe heterostructures, showing a relatively high‐specific surface area. d) XPS spectrum of SnSe_2_/SnSe heterostructures, indicating the coexistence of Sn^4+^ and Sn^2+^. e,f) Raman spectrum and XRD pattern of SnSe_2_/SnSe heterostructures. g) Low‐magnification TEM image of SnSe_2_/SnSe. h–j) Corresponding EDS mapping images of Sn, Se, and C. The uniform element distribution suggests high crystalline quality. k) Quantified elemental analysis of SnSe_2_/SnSe heterostructures. l) Atomic‐resolution HAADF‐STEM image of SnSe_2_/SnSe, showing a sharp interface between SnSe_2_ and SnSe.

Scanning electron microscopy (SEM) measurements are performed on as‐grown SnSe_2_/SnSe to visualize the morphology and microstructure. Interestingly, a unique hierarchical nanoflower morphology is obviously observed, different from the flat feature of single‐phase SnSe_2_ (**Figure** **2**a,b and Figure S1a, Supporting Information). Accordingly, a much higher specific surface area of SnSe_2_/SnSe heterostructures (≈32.1 m^2^ g^−1^) than that of single‐phase SnSe_2_ nanosheets (≈12.3 m^2^ g^−1^) is definitely confirmed by the nitrogen adsorption/desorption isotherms, as shown in Figure 2c and Figure S1b—d, Supporting Information. In a word, the unique hierarchical nanoflower structure of SnSe_2_/SnSe increases the contact efficiency between electrode and electrolyte, which contributes to the formation of a solid electrolyte interface (SEI) layer, and improves the specific capacity and cyclic stability.

X‐ray photoelectron spectroscopy (XPS) is a powerful technology for detecting the chemical states and element compositions and is thus used for characterizing the as‐grown samples (Figure 2d and Figure S2a,b, Supporting Information). The binding energies at ≈492.7 and ≈484.4 eV are attributed to Sn^2+^, while ≈494.7 and ≈485.1 eV are assigned to Sn^4+^, highly indicative of the formation of SnSe_2_/SnSe heterostructures. Furthermore, the content ratio of SnSe:SnSe_2_ in SnSe_2_/SnSe heterostructures is determined to be 0.16:1. The C–C peak of reduced graphene oxide (rGO) is also obtained at ≈282.6 eV (Figure S2c, Supporting Information). The rGO is added to the solution during the synthesis process to further stabilize the cycle life of the lithium‐ion battery. The carbon contents of SnSe_2_/SnSe and SnSe_2_ are calculated to be ≈7.3% and ≈12.7%, respectively (Figure S3, Supporting Information). Raman spectroscopy is also performed to further determine the evolution of SnSe_2_/SnSe heterostructures (Figure 2e and Figure S4, Supporting Information), where the characteristic peaks at ≈70.0 and ≈180.8 cm^−1^ are belonging to A_1g_ and E_2g_ modes of SnSe and SnSe_2_, respectively. The crystalline structure of as‐grown SnSe_2_/SnSe is probed by X‐ray diffraction (XRD). As shown in Figure 2f, the diffraction peaks are ascribed to SnSe_2_ and SnSe, respectively, reconfirming the formation of SnSe_2_/SnSe heterostructures. However, the single‐phase SnSe_2_ nanosheets are obtained as the TOA is replaced by the tetradecylamine (TDA) (Figure S5, Supporting Information). Such results indicate that the TOA‐assisted wet chemical method is beneficial to synthesize SnSe_2_/SnSe heterostructures.

The atomic structures and crystalline qualities of SnSe_2_/SnSe heterostructures are further investigated by transmission electron microscopy (TEM) and high‐angle annular dark‐field scanning transmission electron microscopy (HAADF‐STEM). The low‐magnification TEM image of SnSe_2_/SnSe in Figure 2g presents a perfect hierarchical nanoflower structure, different from the flat feature of single‐phase SnSe_2_ (Figure S6a, Supporting Information). Energy dispersive spectroscopy (EDS) measurements are then carried out to determine the chemical constitutions and their distributions (Figure 2h−j and Figure S6c,d, Supporting Information). The uniform color contrast suggests the high crystalline quality of the obtained SnSe_2_/SnSe heterostructures. The quantified elemental analysis in Figure 2k shows that the atomic ratio of Sn:Se is 29:17, highly indicative of the formation of mixture phases. Notably, in the absence of TOA, single‐phase SnSe_2_ nanosheets are synthesized as verified by the EDS result in Figure S7, Supporting Information. The atomic resolution HAADF‐STEM image in Figure 2l presents a sharp interface between SnSe_2_ and SnSe. The lattice spaces of ≈0.33 and ≈0.41 nm are consistent with the (100) and (101) planes of SnSe_2_ and SnSe, respectively. In short, SnSe_2_/SnSe heterostructures are successfully synthesized by the TOA‐assisted wet chemical method, which provides a platform for exploring electrochemical energy storage applications.

**Figure 3:**
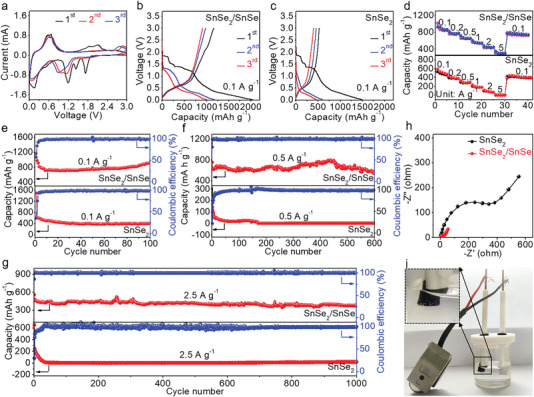
Electrochemical performances of SnSe_2_/SnSe heterostructures. a) CV curves of SnSe_2_/SnSe heterostructures in the first three cycles with the scan rate of 0.1 mV s^−1^. b,c) Charging/discharging curves of SnSe_2_/SnSe heterostructures and single‐phase SnSe_2_ nanosheets in the first three cycles at the current density of 0.1 A g^−1^. d) Corresponding rate capabilities at different current densities. e,f) Cycling performances of SnSe_2_/SnSe heterostructures and single‐phase SnSe_2_ nanosheets at the current densities of 0.1 and 0.5 A g^−1^, respectively. g) Long cycle performances of SnSe_2_/SnSe heterostructures and single‐phase SnSe_2_ nanosheets at a high current density of 2.5 A g^−1^. h) EIS curves of SnSe_2_/SnSe heterostructures and single‐phase SnSe_2_ nanosheets. i) Electrocatalytic HER of SnSn_2_/SnSe by using the lithium‐ion battery composed of SnSe_2_/SnSe anode as the power source.

The electrochemical performances of SnSe_2_/SnSe heterostructures are examined in a half‐cell configuration by using Li foil and SnSe_2_/SnSe as the counter and working electrodes, respectively. **Figure** **3**a and Figure S8, Supporting Information present the cyclic voltammetry (CV) curves of SnSe_2_/SnSe heterostructures and single‐phase SnSe_2_ nanosheets for the first three cycles at the scan rate of 0.1 mV s^−1^. During the first cycle cathodic scan, a sharp peak appeared at ≈1.18 V, which is corresponding to the intercalation of lithium‐ion and the translation of Sn/Li_2_Se. For the following cycles, the peaks at ≈1.59 and ≈1.44 V disappeared, indicative of the decomposing of LiPF_6_ electrolyte and the formation of an irreversible SEI layer. Meanwhile, the alloying and dealloying of Li_x_Sn are convinced by the appearance of a peak at ≈0.51 V and the oxidation peaks at ≈0.80 and ≈0.81 V. The oxidation peak is obtained at ≈1.83 V, indicating the formation of SnSe_2_/SnSe heterostructures. Notably, the subsequent two cycle curves almost overlap, suggestive of the stable surface structure and excellent electrochemical reversibility of SnSe_2_/SnSe. Figure 3b,c reveals the galvanostatic charging/discharging curves of SnSe_2_/SnSe heterostructures and single‐phase SnSe_2_ nanosheets for the first three cycles at the current density of 0.1 A g^−1^. In the first cycle, the discharging and charging capacities of SnSe_2_/SnSe heterostructures are achieved to be ≈2036.0 and ≈1184.0 mAh g^−1^, respectively, with an initial Coulombic efficiency (ICE) of ≈54%, much higher than that of single‐phase SnSe_2_ nanosheets (≈35%). The limited ICE is mainly concerned with the formation of the SEI layer and the irreversible reaction on the electrode surfaces.

The rate capacities of SnSe_2_/SnSe heterostructures and single‐phase SnSe_2_ nanosheets are evaluated at different current densities (Figure 3d). For example, as the current density increases from 0.1 to 0.2 A g^−1^, SnSe_2_/SnSe heterostructures display the reversible capacities of ≈842.6 and ≈764.6 mAh g^−1^, respectively, much higher than those of single‐phase SnSe_2_ nanosheets (≈455.6 and ≈373.5 mAh g^−1^). More interestingly, a reversible capacity of ≈295.8 mAh g^−1^ is obtained even at a high current density of 5.0 A g^−1^, indicative of the excellent rate performance of SnSe_2_/SnSe heterostructures. In order to explore the influence of the ratio of SnSe:SnSe_2_ in SnSe_2_/SnSe heterostructures on the lithium‐ion battery performances, the corresponding electrochemical measurements are then performed. Interestingly, by increasing the ratio from 0.09:1 to 0.16:1, the capacities of SnSe_2_/SnSe heterostructures are increased accordingly. Nevertheless, further increasing the ratios to 0.4:1 and 1.78:1, the capacities are decreased, and thus the optimal ratio is determined to be 0.16:1 (Figures S9 and S10, Supporting Information). The morphologies of SnSe_2_/SnSe heterostructures with different ratios are presented in Figure S11, Supporting Information, where traditional nanosheets with flat features are obtained for the ratios of 0.09:1, 0.1:1, 0.4:1, and 1.78:1, completely different from the hierarchical nanoflower of SnSe_2_/SnSe with the optimal ratio of 0.16:1. Such a unique morphology increases the contact area between electrode and electrolyte, buffers the volume expansion, and improves the cyclic stability.

Furthermore, the cycling performances at different current densities are also tested to determine the stability of electrodes. SnSe_2_/SnSe heterostructures present a reversible capacity of ≈916.0 mAh g^−1^ at 0.1 A g^−1^ with a Coulombic efficiency of ≈100%, and the capacity can be maintained to ≈911.4 mAh g^−1^ even after 100 cycles, implying its outstanding durability (Figure 3e). An upward trend of discharging capacity is observed during the cycling processes, possibly due to the increased roughness and porosity. Meanwhile, the conversion reaction has also occurred on the surfaces of SnSe_2_/SnSe, which increases the contact area between electrode and electrolyte and thus improves the discharging capacity. Nevertheless, the reversible capacity of single‐phase SnSe_2_ is only obtained to be ≈404.8 mAh g^−1^ after 100 cycles, suggesting its low stability. As shown in Figure 3f, upon cycling at 0.5 A g^−1^, a capacity of ≈536.4 mAh g^−1^ is harvested after 600 cycles for SnSe_2_/SnSe, much higher than that of single‐phase SnSe_2_ (≈2.8 mAh g^−1^). Even at an elevated current density of 2.5 A g^−1^, the SnSe_2_/SnSe heterostructures still deliver a favorable cycling performance as shown in Figure 3g. In addition, the electrochemical measurements of single‐phase SnSe are also performed, with the results shown in Figure S12, Supporting Information. The inferior lithium storage performances are obtained possibly due to the relatively low electronic conductivity and poor structure stability. Notably, the morphology of single‐phase SnSe is revealed in Figure S13, Supporting Information and the flat/thick nanosheets are observed, which limit the ion transport efficiency and hinder the improvement of cyclic stability.

The morphologies and structures of SnSe_2_/SnSe are still retained even after 100 cycles at 0.1 A g^−1^, reconfirming the high stability (Figures S14 and S15, Supporting Information). The unique hierarchical nanoflower structure of SnSe_2_/SnSe provides abundant transport channels and contributes to the improvement of cyclic stability. In addition, the ultralow charge‐transfer resistance (≈24 Ω) is also extracted from the electrochemical impedance spectra (ESI) of SnSe_2_/SnSe, much smaller than that of single‐phase SnSe_2_ (≈346 Ω), indicative of the fast charge transfer (Figure 3h). The EIS curves of SnSe_2_/SnSe heterostructures and single‐phase SnSe_2_ after cycles are also obtained and shown in Figure S16. Notably, the lithium‐ion battery composed of SnSe_2_/SnSe anode can be used as the power source to catalyze the hydrogen evolution reaction (HER) with SnSn_2_/SnSe as the working electrode (Figure 3i and Movie S1, Supporting Information) and lighten a light‐emitting diode bulb (Figure S17, Supporting Information). To gain insight into the electrochemical kinetics of SnSe_2_/SnSe heterostructures, the CV analysis is performed with the scan rates ranging from 0.2 to 1.6 mV s^−1^ (Figure S18a, Supporting Information). The pseudocapacitive contributions are calculated to be ≈31%, ≈37%, ≈43%, ≈53%, ≈56%, and ≈66% at the scan rates of 0.2, 0.4, 0.6, 0.8, 1.2, and 1.4 mV s^−1^, respectively (Figure S18b,c, Supporting Information). This readily suggests that the capacitive‐controlled lithium storage in SnSe_2_/SnSe heterostructures is predominant, especially at a high scan rate. The contrastive lithium‐ion battery performances of SnSe_2_/SnSe with other Sn‐based compounds are shown in Table S1, Supporting Information.

**Figure 4:**
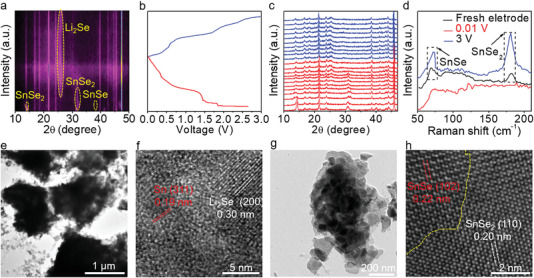
Structure evolution of SnSe_2_/SnSe during the charging/discharging processes. a) In situ XRD patterns of SnSe_2_/SnSe for the first cycle. b,c) Corresponding charging/discharging curves and in situ XRD patterns. d) Raman spectra of SnSn_2_/SnSe at different stages. e,f) Low‐magnification and atomic‐resolution TEM images of SnSe_2_/SnSe at the end of discharging process. g,h) Low‐magnification and atomic‐resolution TEM images of SnSe_2_/SnSe at the end of the charging process.

In situ XRD, Raman spectroscopy, and TEM measurements are then performed to further explore the internal mechanism. The in situ half‐cell is discharged from the open‐circuit voltage (OCP) to ≈0.01 V and then charged from ≈0.01 to ≈3 V (Figure 4a−c). Along with the discharging process, the XRD peaks of SnSe_2_ at ≈14.6° and SnSe at ≈37.9° gradually disappeared, suggestive of the lithium‐ion intercalation. The appearance of ≈25.0° and ≈30.7° characteristic peaks indicates the formation of Li_2_Se and Sn, as well as the decomposition of SnSe_2_/SnSe. Notably, the XRD peak of Li_2_Se is retained throughout the charging processes, nevertheless, the characteristic peaks of SnSe_2_/SnSe are not appeared, possibly due to its deteriorative crystallinity. Even so, the atomic structures, hierarchical nanoflower morphologies, and interfaces of SnSe_2_/SnSe are still maintained, as verified by Raman, TEM, and SEM results. Similar phenomena are demonstrated in other materials.^[^ ^49^, ^50^, ^51^, ^52^ ^]^ In addition, the charging/discharging processes of single‐phase SnSe_2_ are also investigated by the ex situ XRD at different voltages, as shown in Figure S19, Supporting Information. According to the in situ XRD results, the chemical reactions of SnSe_2_/SnSe during the charging/discharging processes can be summarized as follows:

- Initial lithiation processes:
(1) $${\mathrm{SnSe}}_{2}+4Li^{+}+4e^{-}\to \mathrm{Sn}+2Li_{2}\mathrm{Se}$$
(2) $$\mathrm{Sn}+x{\mathrm{Li}}^{+}+xe^{-}\to {\mathrm{Li}}_{x}\mathrm{Sn}\left(x\leq 4.4\right)$$
- Subsequent delithiation/lithiation processes:
(3) $${\mathrm{Li}}_{x}\mathrm{Sn}+x{\mathrm{Li}}^{+}+xe^{-}↔\mathrm{Sn}\left(x\leq 4.4\right)$$
(4) $$\mathrm{Sn}+2Li_{2}\mathrm{Se}+4Li^{+}+4e^{-}↔{\mathrm{SnSe}}_{2}$$
(5) $${\mathrm{Li}}_{2}\mathrm{Se}+2Li^{+}+2e^{-}↔\mathrm{Se}$$

Raman characterizations are then employed to analyze the structure evolution of SnSe_2_/SnSe heterostructures. Before the discharging process, the characteristic peaks of SnSe_2_ and SnSe are obviously observed, as shown in **Figure** **4**d. However, after finishing the discharging process, the corresponding Raman peaks disappeared, which indicates the complete decomposition of SnSe_2_/SnSe. Interestingly, after charging to ≈3 V, the characteristic peaks of SnSe_2_ and SnSe are observed again, suggesting the reappearance of SnSe_2_/SnSe heterostructures. In addition, the hierarchical nanoflower morphology of SnSe_2_/SnSe heterostructures is maintained during the charging/discharging processes, as convinced by the TEM results in Figure 4e,g. At the end of discharging process, Li_2_Se and Sn are obtained with the interplanar spaces of ≈0.30 and ≈0.19 nm, respectively (Figure 4f). After charging to 3.0 V, SnSe_2_/SnSe heterostructures reappeared with a sharp interface, indicative of extraordinary structural stability (Figure 4h).

**Figure 5:**
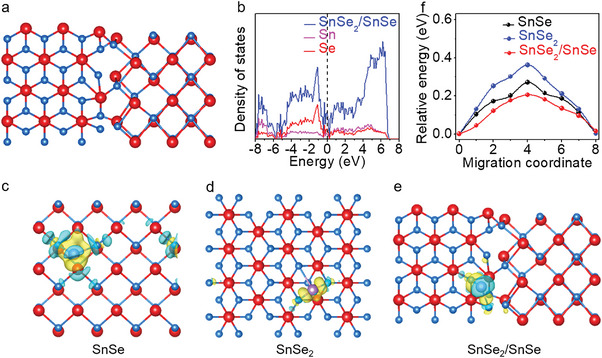
DFT calculated the band structure of SnSe_2_/SnSe and the diffusion barrier of the lithium atom. a) Optimized atomic structure of SnSe_2_/SnSe. b) Corresponding band structure of SnSe_2_/SnSe. c−e) Differential charge densities of SnSe, SnSe_2_, and SnSe_2_/SnSe. f) Migration energy of lithium atom from one adsorption site to the adjacent adsorption site.

To further understand the internal mechanism of such excellent lithium storage performances for SnSe_2_/SnSe heterostructures, the density functional theory (DFT) calculations are thus carried out (**Figure** **5**a and Figure S20, Supporting Information). Interestingly, a novel metallic feature is obviously observed in SnSe_2_/SnSe (Figure 5b), totally different from the semiconducting properties of SnSe_2_ (≈1.10 eV) and SnSe (≈0.56 eV). A similar phenomenon is also obtained in the previous result.^[^ ^45^ ^]^ The ultrahigh electronic conductivity in SnSe_2_/SnSe heterostructures accelerates the charge transfer and contributes to the enhancement of lithium‐ion battery performances. In addition, the differential charge densities of SnSe, SnSe_2_, and SnSe_2_/SnSe are calculated and revealed in Figure 5c−e, respectively. The accumulation of charges at the interfaces of SnSe_2_/SnSe heterostructures indicates the establishment of a built‐in electric field, which is favorable for migrating lithium atoms. Meanwhile, a much lower migration barrier of lithium atoms on SnSe_2_/SnSe than that on SnSe_2_ and SnSe is also presented in Figure 5f, which suggests the fast reaction kinetics and excellent electrochemical performance. The unique electronic structure of SnSe_2_/SnSe modifies the distribution of electrons and the interfacial effect accelerates the transport of ions, which contributes to the enhancement of lithium‐ion battery performances. In short, to achieve the ultrahigh specific capacity and robust cycling stability of the lithium‐ion battery, the anode materials should possess large specific surface areas and excellent electronic conductivities. The construction of heterostructures provides a perfect approach because of their unique physical properties and interfacial effect. The robust interfaces contribute to the redistribution of electrons and the optimization of electronic structures.^[^ ^53^, ^54^ ^]^

## 3 Conclusion

A facile and high‐throughput wet chemical method is designed to batch synthesis of metallic SnSe_2_/SnSe heterostructures. The unique hierarchical nanoflower morphology increases the specific surface area and contributes to the improvement of cyclic stability. Furthermore, the establishment of a built‐in electric field and metallic feature in SnSe_2_/SnSe enhance the charge transport capability. Accordingly, the remarkable lithium storage performances are demonstrated in SnSe_2_/SnSe, featured with an excellent specific capacity, high rate performance, and outstanding cyclic stability. Such results present a prominent advance toward boosting lithium‐ion battery performances by constructing heterostructures.

## 4 Experimental Section

### Material Syntheses

50 mL OAm and 50 mL TOA were mixed and added in a 250 mL three‐neck flask, and then heated to ≈120 °C for 30 min under N_2_ atmosphere to remove the dissolved water and oxygen. A transparent solution was obtained after cooling down to ≈50 °C. Afterward, ≈1.5 mmol SnCl_4·_5H_2_O, 4 mmol Se powders, and 50 mg rGO were quickly added into the three‐neck flask with vigorous stirring until a dark solution was observed. Subsequently, the obtained solution was heated to ≈300 °C at the rate of ≈10 °C min^−1^ and kept for 4 h to achieve SnSe_2_/SnSe heterostructures. After naturally cooling down to ≈70 °C, 10 mL of tri‐n‐octylphosphine (TOP) was slowly injected into the solution to remove the excess Se powders. After the reaction for 1 hour, the black precipitate was centrifuged and washed with the hexane several times, and then dried in a vacuum oven at ≈60 °C. In order to improve the crystalline quality, as‐grown SnSe_2_/SnSe heterostructures were annealed at ≈400 °C for 1 h at the rate of ≈5 °C min^−1^ under Ar atmosphere.

### Material Characterizations

The obtained SnSe_2_/SnSe heterostructures were systematically characterized by SEM (Hitachi S‐4800, with the acceleration voltage of ≈5 kV), XPS (ESCALAB250Xi, with the excitation source of Mg K*α*), XRD (Rigaku Smartlab SE, using Cu K*α* radiation in the 2*θ* range of 10°−90°), Raman spectroscopy (Renishaw, with the excitation light of ≈532 nm), TGA (Mettler‐Toledo TGA/DSC 3+), and TEM (JEOL JEM‐F200 and JEM‐NEOARM, with the acceleration voltage of ≈200 kV). The specific surface area was obtained by using the Brunauer–Emmett–Teller (BET) method.

### Electrochemical Measurements

The obtained SnSe_2_/SnSe heterostructures were mixed with acetylene black and polyvinylidene fluoride (PVDF) with a weight ratio of 8:1:1 in n‐methyl‐2‐pyrrolidone (NMP). The loading density of SnSe_2_/SnSe heterostructures on the electrode was set to be ≈0.8 to 1.2 mg cm^−2^. The slurry was cast on Cu foil and then dried for 10 h at ≈120 °C under vacuum. The 2032‐type coin cells were assembled in the Ar‐filled glove box, with Li foil as the counter electrode. 1.0 M LiPF_6_ mixtures with ethylene carbonate/dimethyl carbonate (EC/DMC, 1:1 vol.%) and ≈5 wt% fluoroethylene carbonate were used as the electrolyte. The electrochemical performances of SnSe_2_/SnSe heterostructures and single‐phase SnSe_2_ nanosheets were measured on the LAND CT2001A multi‐channel battery testing system with a voltage window of ≈0.001 to 3.0 V. The CV curves were recorded on a CHI 660D electrochemical workstation at a scan rate of 0.2 mV s^−1^ with a potential interval of ≈0.001 to 3.0 V. EIS was tested by using the CHI 660D electrochemical workstation with a frequency range from 100 kHz to 10 mHz.

### Theoretical Calculations

DFT calculations were performed by using the Vienna ab initio simulation package (VASP) with the projector augmented wave (PAW) method.^[^ ^55^, ^56^ ^]^ The Perdew–Burke–Ernzerhof (PBE) functional for the exchange‐correlation term was used with the projector augmented wave method,^[^ ^57^ ^]^ and the kinetic energy cutoff of electron wave functions was set to be 450 eV. The partial occupancies of Kohn–Sham orbitals were allowed by using the Gaussian smearing method and a width of 0.05 eV. The electronic energy was considered self‐consistent as the energy change was smaller than 10^−5^ eV. A geometry optimization was considered convergent when the force change was smaller than 0.03 eV Å^−1^. The DFT‐D3 method was used to describe the dispersion interaction. The equilibrium lattice constants of the surface were optimized when using a 2 × 2 × 1 Monkhorst–Pack *k*‐point grid for Brillouin zone sampling. The climbing image‐nudged elastic band methods were employed to calculate the migration barrier of lithium atoms.

## Conflict of Interest

The authors declare no conflict of interest.

## Supporting information

Supporting Information

Supplemental Movie 1

## Data Availability

The data that support the findings of this study are available from the corresponding author upon reasonable request.
