# Supplementary material for: Interfacial Coupling SnSe2/SnSe Heterostructures as Long Cyclic Anodes of Lithium‐Ion Battery
Source: Adv Sci (Weinh). 2022 Nov 18;10(2):2204671. doi: 10.1002/advs.202204671 (PMC9839860; doi:10.1002/advs.202204671)
Supplement: Supplementary file 1 — Supporting Information [file ADVS-10-2204671-s001.pdf]

Supporting Information

Interfacial Coupling $SnSe_2$/SnSe Heterostructures as Long Cyclic Anodes of Lithium-Ion Battery

*Wang Feng, Xia Wen, Yuzhu Wang, Luying Song, Xiaohui Li, Ruofan Du, Junbo Yang, Hui Li, Jun He, and Jianping Shi**

W. Feng, X. Wen, Y. Z. Wang, L. Y. Song, X. H. Li, R. F. Du, J. B. Yang, H. Li, Prof. J. P. Shi
The Institute for Advanced Studies, Wuhan University, Wuhan 430072, China
E-mail: jianpingshi@whu.edu.cn

Prof. J. He
Key Laboratory of Artificial Micro- and Nano-structures of Ministry of Education, School of Physics and Technology, Wuhan University, Wuhan 430072, China

Keywords: $SnSe_2$/SnSe heterostructures, interfacial coupling, lithium-ion battery, long cyclic stability, high specific capacity

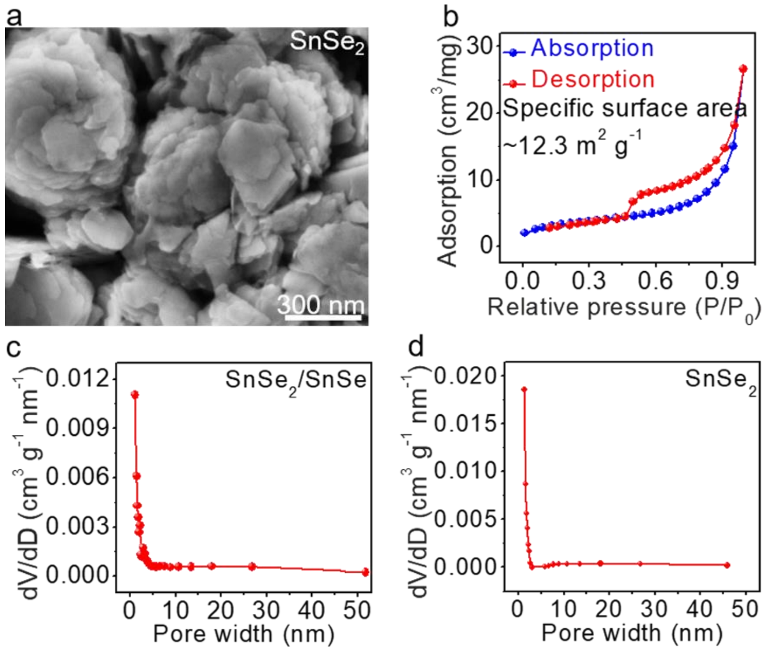

Figure S1. Morphologies and specific surface areas of single-phase $SnSe_2$ nanosheets. (a) SEM image of single-phase $SnSe_2$ nanosheets, showing the flat feature. (b) Nitrogen adsorption/desorption isotherms of single-phase $SnSe_2$ nanosheets. (c,d) Pore size distributions of $SnSe_2$/SnSe heterostructures and single-phase $SnSe_2$ nanosheets.

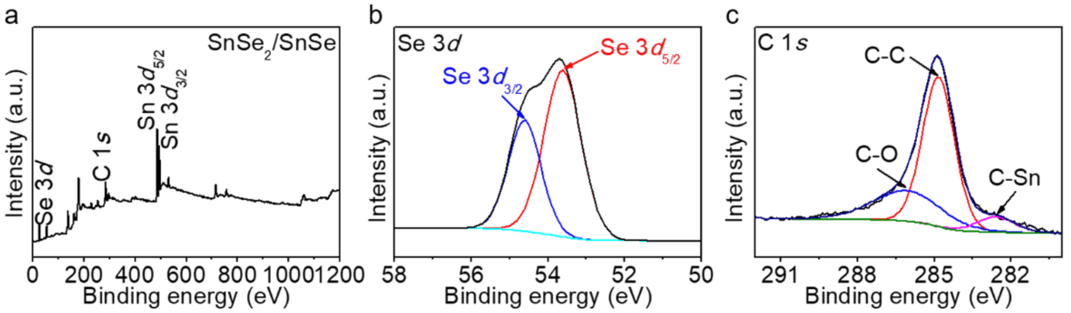

Figure S2. XPS characterizations of as-grown $SnSe_2$/SnSe heterostructures. (a) XPS spectrum acquired over a wide range of binding energies (0~1200 eV). (b) XPS spectrum of Se 3*d*. The binding energies of 3$d_{3/2}$ and 3$d_{5/2}$ are located at ~53.6 and ~54.6 eV, respectively. (c) XPS spectrum of C 1*s*. The C-C characteristic peak is obtained at ~282.6 eV.

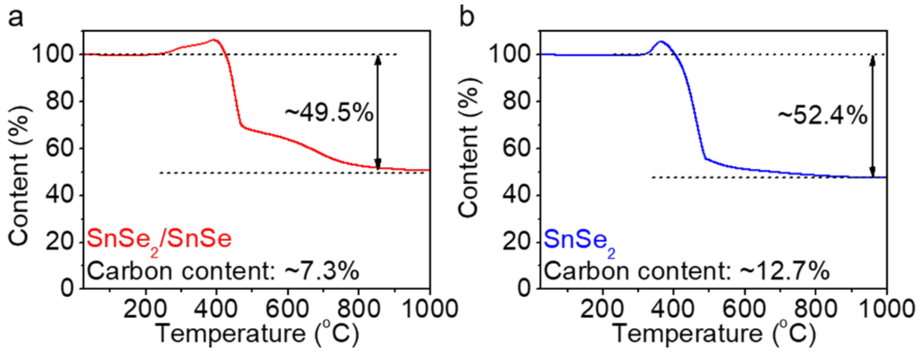

Figure S3. Thermogravimetric analysis (TGA) curves of $SnSe_2$/SnSe heterostructures and single-phase $SnSe_2$ nanosheets. The carbon contents of $SnSe_2$/SnSe and $SnSe_2$ are calculated to be ~7.3% and ~12.7%, respectively.

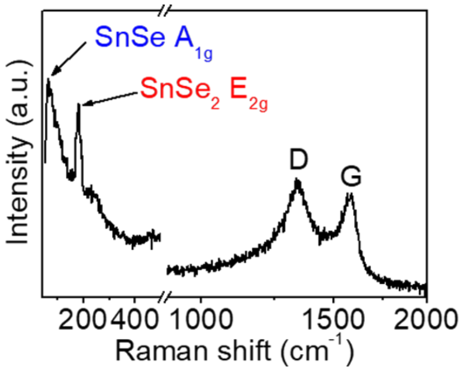

Figure S4. Raman spectrum of as-grown $SnSe_2$/SnSe heterostructures. The characteristic peaks of $SnSe_2$ (E_{2g}) and SnSe (A_{1g}) are obviously observed at ~70.0 and ~180.8 cm^{-1}, respectively.

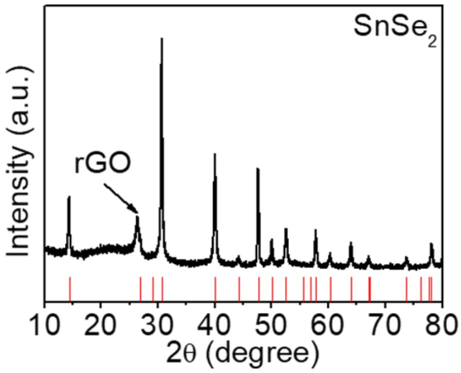

Figure S5. XRD pattern of as-grown single-phase $SnSe_2$ nanosheets. The sharp characteristic peaks indicate the high crystalline quality of the obtained samples.

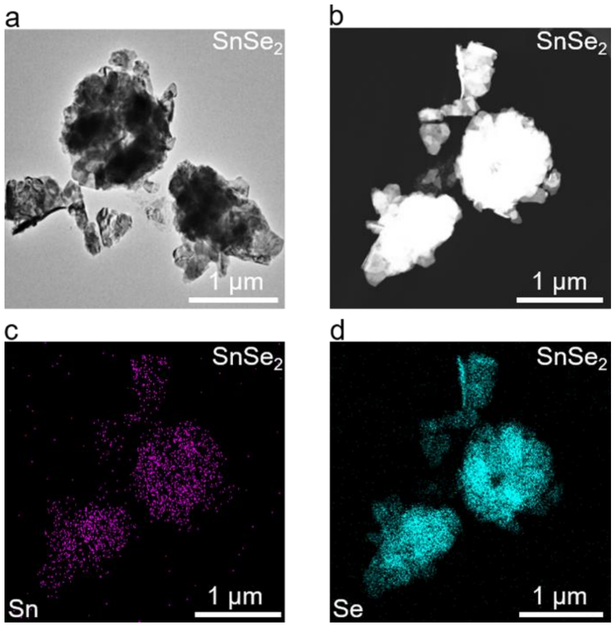

Figure S6. Morphologies and elemental distributions of single-phase $SnSe_2$ nanosheets. (a) SEM image of single-phase $SnSe_2$ nanosheets. (b) Low-magnification TEM image of single-phase $SnSe_2$ nanosheets. (c,d) Corresponding EDS mapping images of Sn and Se. The uniform element distribution suggests the high crystalline quality.

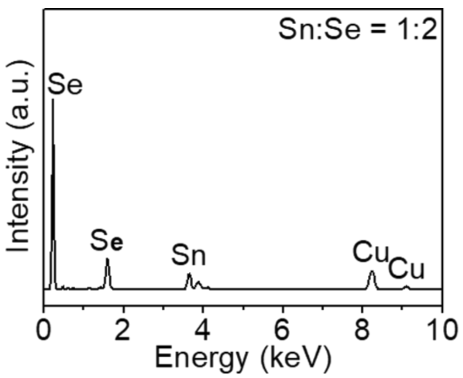

Figure S7. Quantified elemental analysis of single-phase $SnSe_2$ nanosheets. The atomic ratio of Sn:Se is calculated to be 1:2.

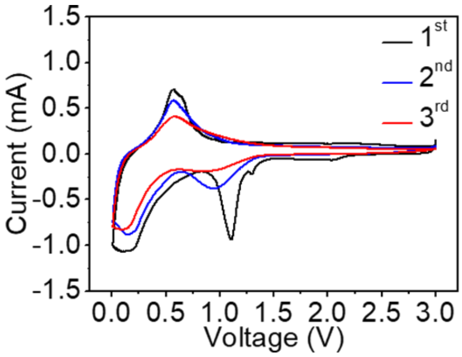

Figure S8. CV curves of single-phase $SnSe_2$ nanosheets of the first three cycles with a scan rate of 0.1 mV s^{-1}.

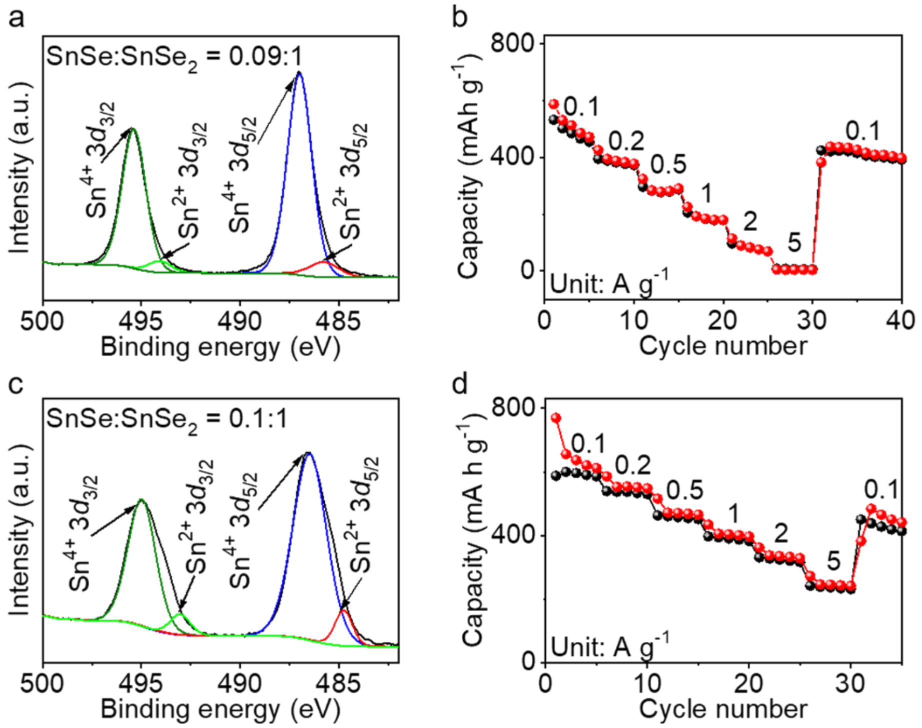

Figure S9. The influence of ratio of SnSe:$SnSe_2$ in $SnSe_2$/SnSe heterostructures on the lithium storage performances. (a,c) XPS spectra of $SnSe_2$/SnSe with the ratios of 0.09:1 and 0.1:1. (b,d) Corresponding rate capabilities at different current densities.

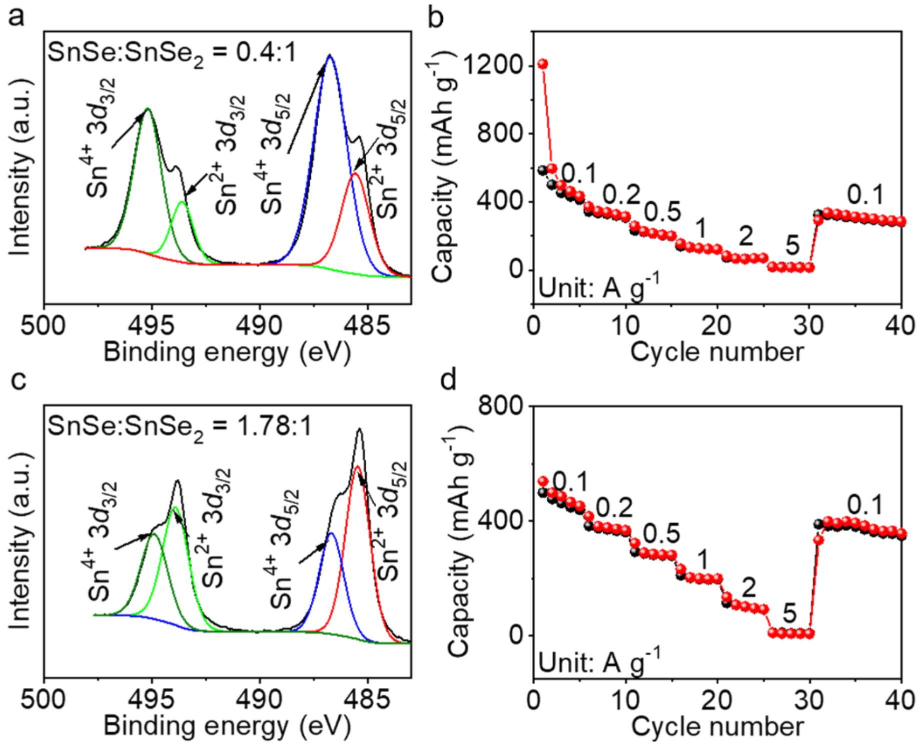

Figure S10. The influence of ratio of SnSe:$SnSe_2$ in $SnSe_2$/SnSe heterostructures on the lithium storage performances. (a,c) XPS spectra of $SnSe_2$/SnSe with the ratios of 0.4:1 and 1.78:1. (b,d) Corresponding rate capabilities at different current densities.

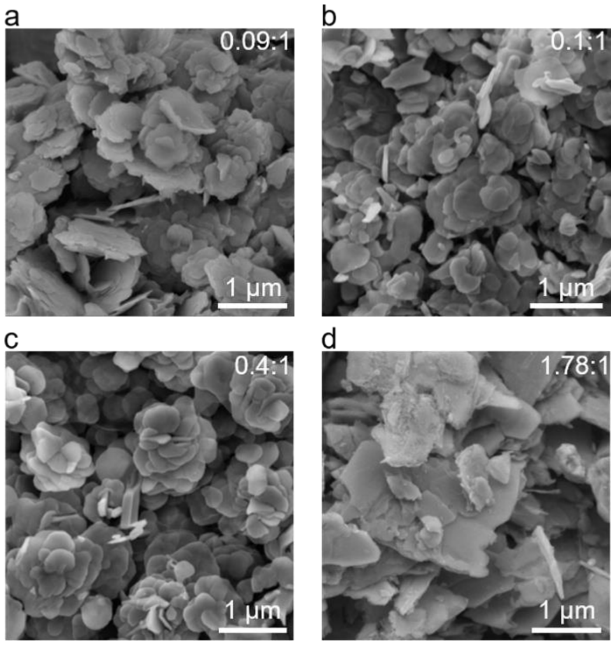

Figure S11. Morphology analyses of $SnSe_2$/SnSe heterostructures with different ratios. (a−d) SEM images of $SnSe_2$/SnSe heterostructures with the ratios of 0.09:1, 0.1:1, 0.4:1, and 1.78:1, respectively.

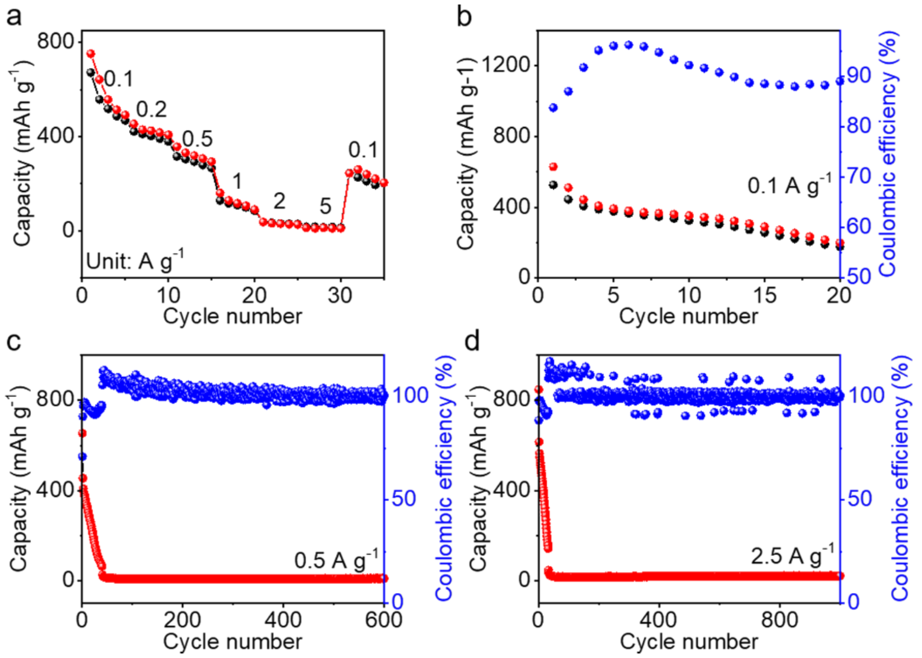

Figure S12. Lithium storage performances of single-phase SnSe. (a) Rate capabilities at different current densities. (b–d) Cycling performances of single-phase SnSe at the current densities of 0.1, 0.5, and 2.5 A g^{-1}, respectively.

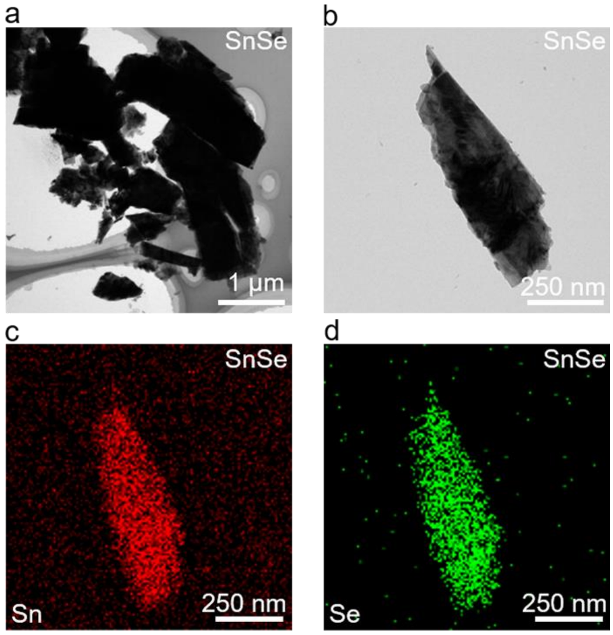

Figure S13. Morphologies and elemental distributions of single-phase SnSe. (a,b) Low-magnification TEM images of single-phase SnSe nanosheets. (c,d) Corresponding EDS mapping images of Sn and Se.

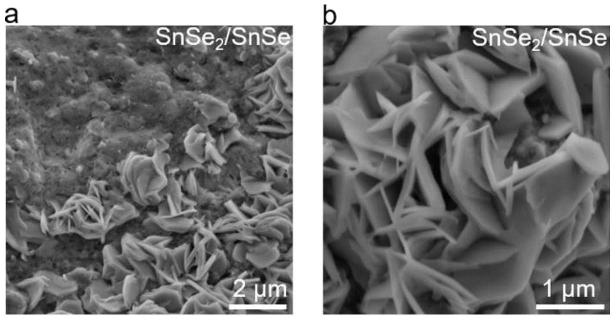

Figure S14. SEM images of $SnSe_2$/SnSe heterostructures. The morphologies and structures of $SnSe_2$/SnSe are maintained after 100 cycles at the current density of 0.1 A g^{-1}, indicating the robust stability.

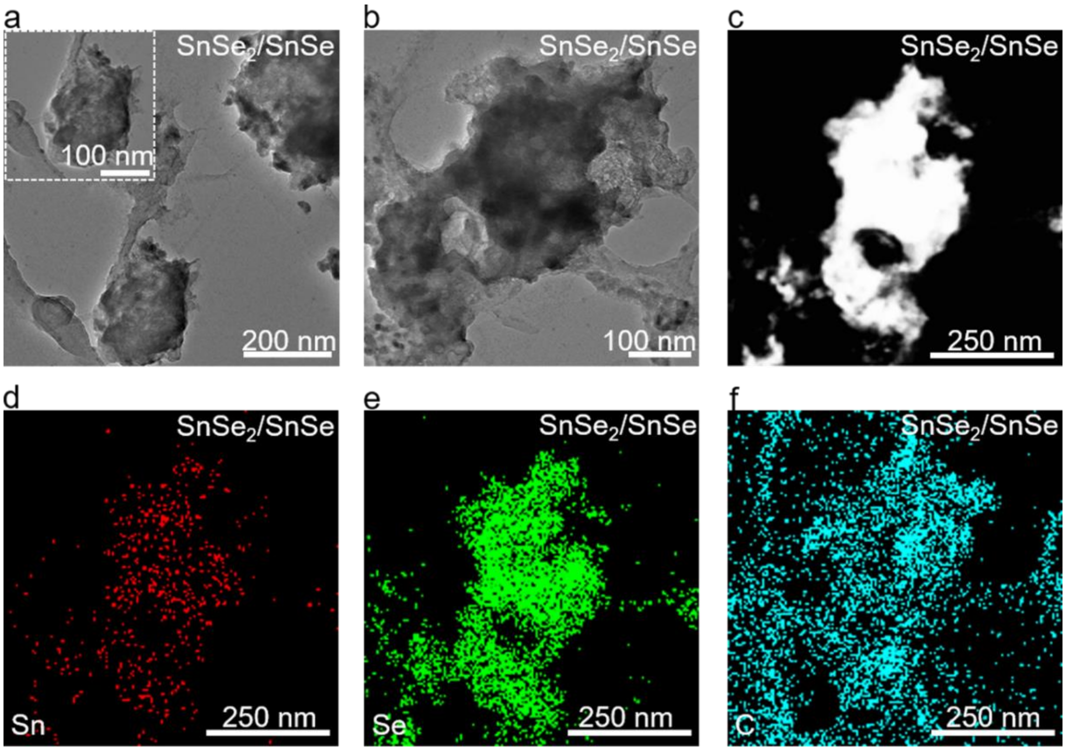

Figure S15. Morphologies of $SnSe_2$/SnSe heterostructures after the cycling tests. (a–c) Low-magnification TEM images of $SnSe_2$/SnSe heterostructures after 100 cycles at the current density of 0.1 A g^{-1}. (d–f) Corresponding EDS mapping images of Sn, Se, and C.

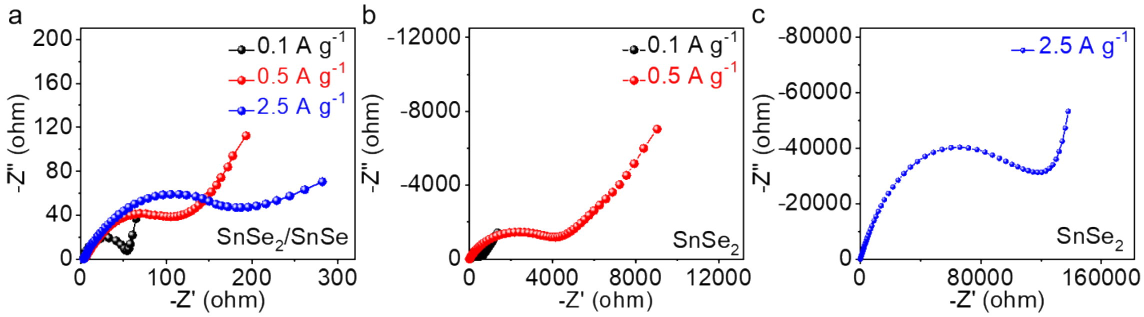

Figure S16. The ultrafast charge transfer in $SnSe_2$/SnSe heterostructures. (a) EIS curves of $SnSe_2$/SnSe heterostructures after 100 cycles at 0.1 A g^{-1}, 600 cycles at 0.5 A g^{-1}, and 1000 cycles at 2.5 A g^{-1}. (b,c) EIS curves of single-phase $SnSe_2$ after 100 cycles at 0.1 A g^{-1}, 600 cycles at 0.5 A g^{-1}, and 1000 cycles at 2.5 A g^{-1}.

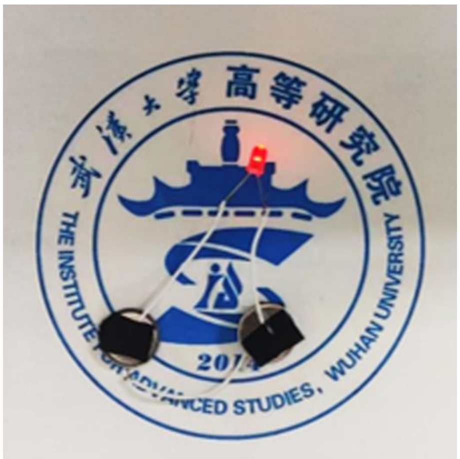

Figure S17. Photograph of the lithium-ion battery composed of $SnSe_2$/SnSe anode powering an LED bulb.

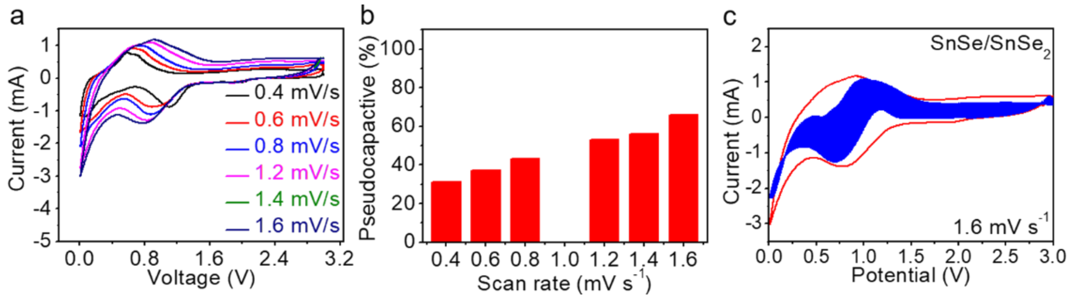

Figure S18. Electrochemical kinetics of $SnSe_2$/SnSe heterostructures for lithium-ion storage. (a) CV curves of $SnSe_2$/SnSe heterostructures at different scan rates. (b) Pseudocapacitive contribution of $SnSe_2$/SnSe heterostructures at different scan rates. (c) CV curve with the capacitive contribution at a scan rate of 1.6 mV S^{-1}.

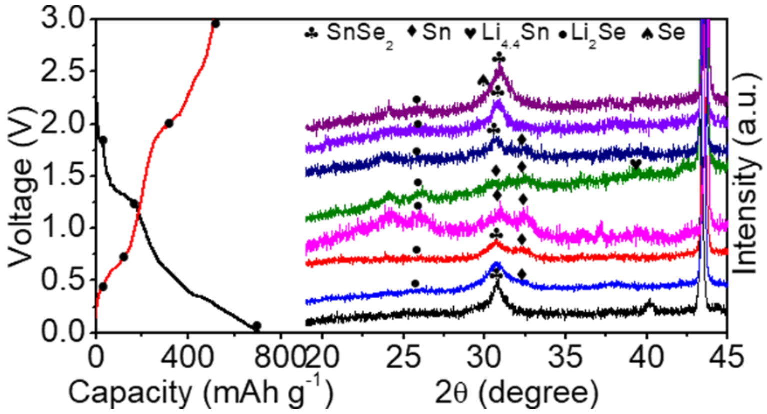

Figure S19. Ex-situ XRD characterizations of single-phase $SnSe_2$ nanosheets at different voltages during the charging/discharging processes.

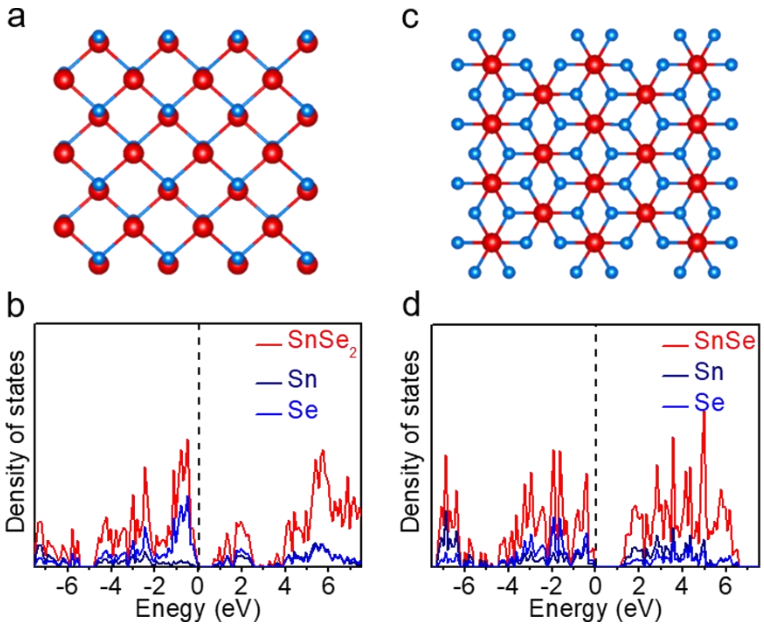

Figure S20. DFT calculated the band structures of $SnSe_2$ and SnSe. (a,c) Optimized atomic structures of $SnSe_2$ and SnSe. (b,d) Band structures of $SnSe_2$ and SnSe.

Table S1. The contrastive lithium-ion battery performances of $SnSe_2$/SnSe with the other Sn-based compounds

Materials	Capacity (mAh g^{-1})	Cycle number	Ref.
$SnSe_2$/graphene	490.9@0.1 A g^{-1}	1500@0.1 A g^{-1}	1
$SnSe_2$/carbon nanotubes	457.6@0.1 A g^{-1}	100@0.1 A g^{-1}	2
$SnSe_2$/rGO	778.5@0.05 A g^{-1}	500@0.2 A g^{-1}	3
$SnSe_2$/rGO	402.0@0.1 A g^{-1}	150@0.1 A g^{-1}	4
$SnSe_2$/rGO	640@40 mA g^{-1}	30@40 mA g^{-1}	5
$SnSe_2$/grephene	490.9@0.1 A g^{-1}	202.5@0.5 A g^{-1}	6
SnSe	683.6@0.1 A g^{-1}	100@0.1 A g^{-1}	7
SnSe	787.9@0.1 A g^{-1}	300@0.1 A g^{-1}	8
**$SnSe_2$/SnSe**	**911.4@0.1 A g^{-1}**	**1000@2.5 A g^{-1}**	**This work**

References

[1] H. W. Chen, R. M. Liu, Y. Wu, J. H. Cao, J. Chen, Y. Hou, Y. C. Guo, R. Khatoon, L. X. Chen, Q. H. Zhang, Q. G. He, J. G. Lu, *Chem. Eng. J.* **2021**, *407*, 126973.

[2] H. Chen, B.-E. Jia, X. S. Lu, Y. C. Guo, R. Hu, R. Khatoon, L. Jiao, J. X. Leng, L. Q. Zhang, J. G. Lu, *Chem. Eur. J.* **2019**, *25*, 9973.

[3] Z. X. Huang, B. Liu, D. Z. Kong, Y. Wang, H. Y. Yang, *Energy Stor. Mater.* **2018**, *10*, 92.

[4] T. T. Wang, K. W. Yang, J. Shi, S. R. Zhou, L. W. Mi, H. P. Li, W. H. Chen, *J. Energy. Chem.* **2020**, *46*, 71.

[5] J. Choi, J. Jin, I. G. Jung, J. M. Kim, H. J. Kim, S. U. Son, *Chem. Commun.* **2011**, *47*, 5241.

[6] H. W. Chen, R. M. Liu, Y. Wu, J. H. Cao, J. Chen, Y. Hou, Y. C. Guo, R. Khatoon, L. X Chen, Q. H Zhang, Q. G. He, J. G. Lu, *Chem. Eng. J.* **2021**, *407*, 126973.

[7] Y. P. Chen, Q. L. Yang, P. B. Wu, T. X. Xu, J. Wang, Y. J. Li, *ACS Appl. Nano Mater.* **2021**, *4*, 13010.

[8] W. Wang, P. H. Li, H. Zheng, Q. Liu, F. Lv, J. D. Wu, H. Wang, S. J. Guo, *Small* **2017**, *13*, 1702228.
